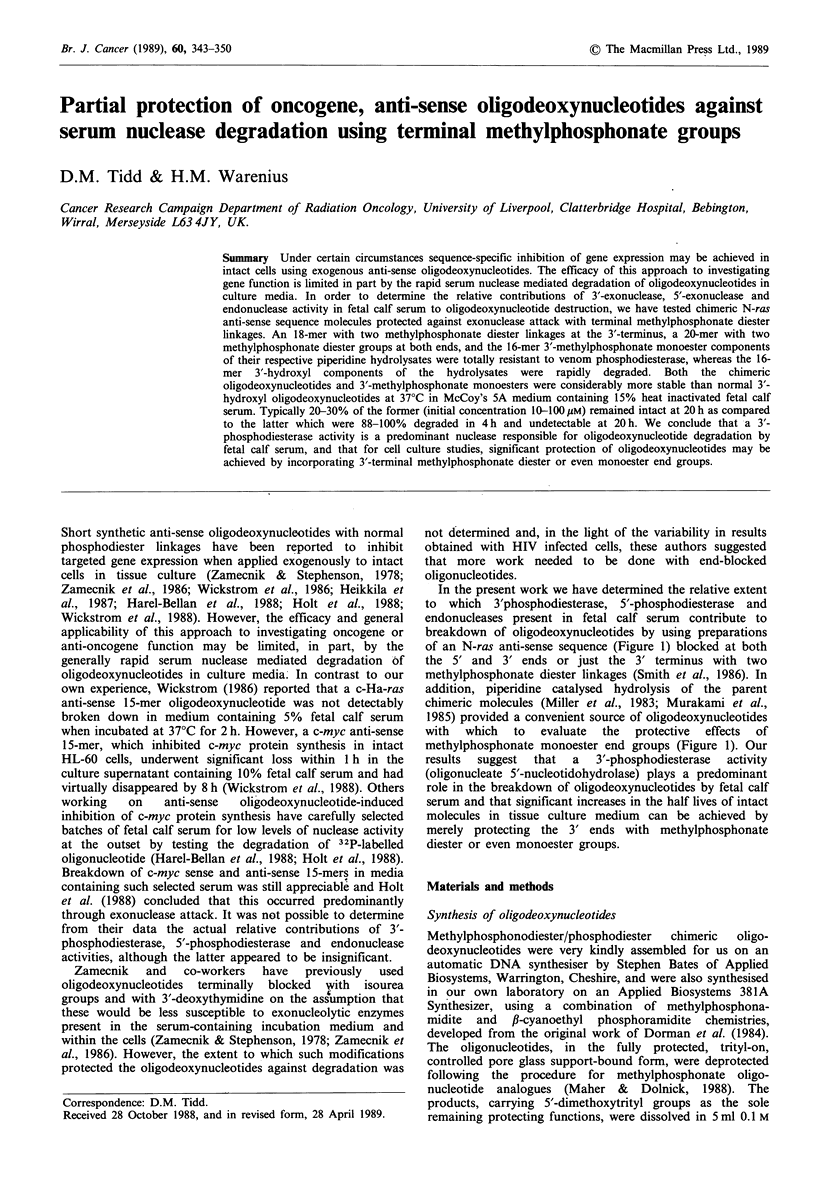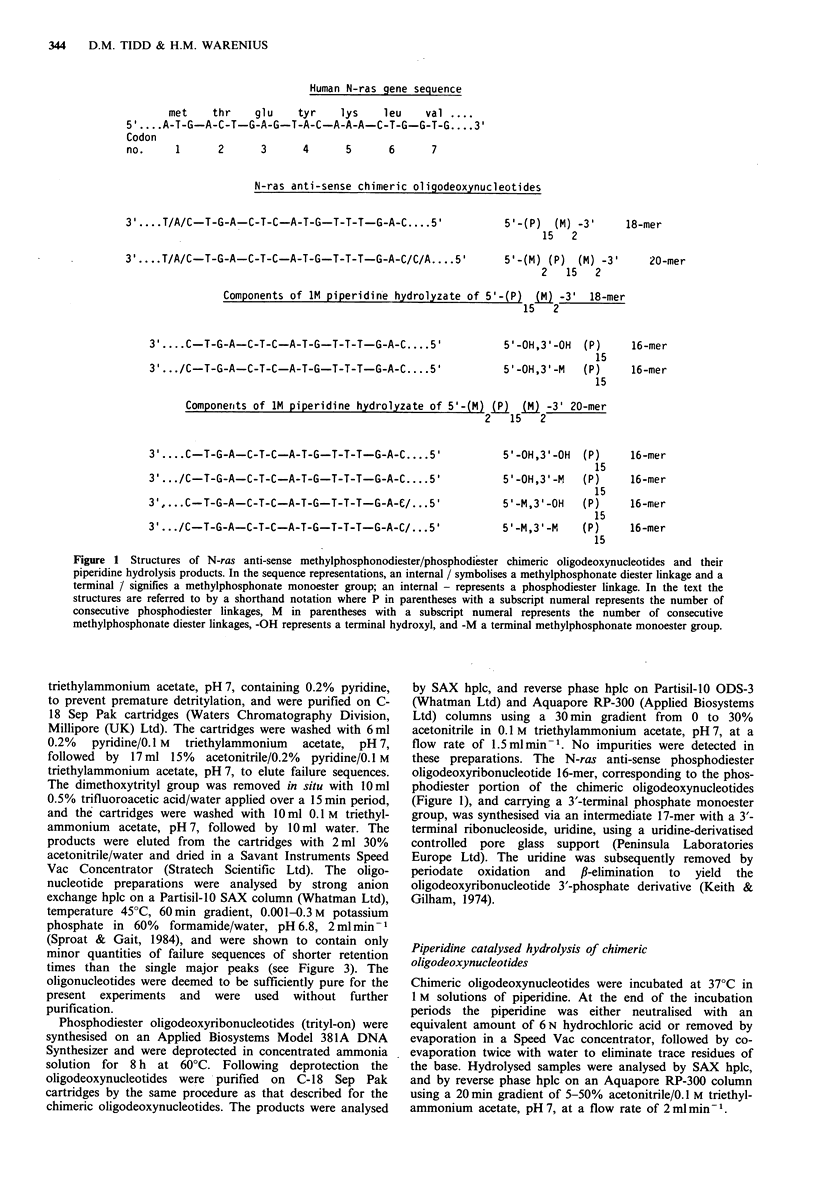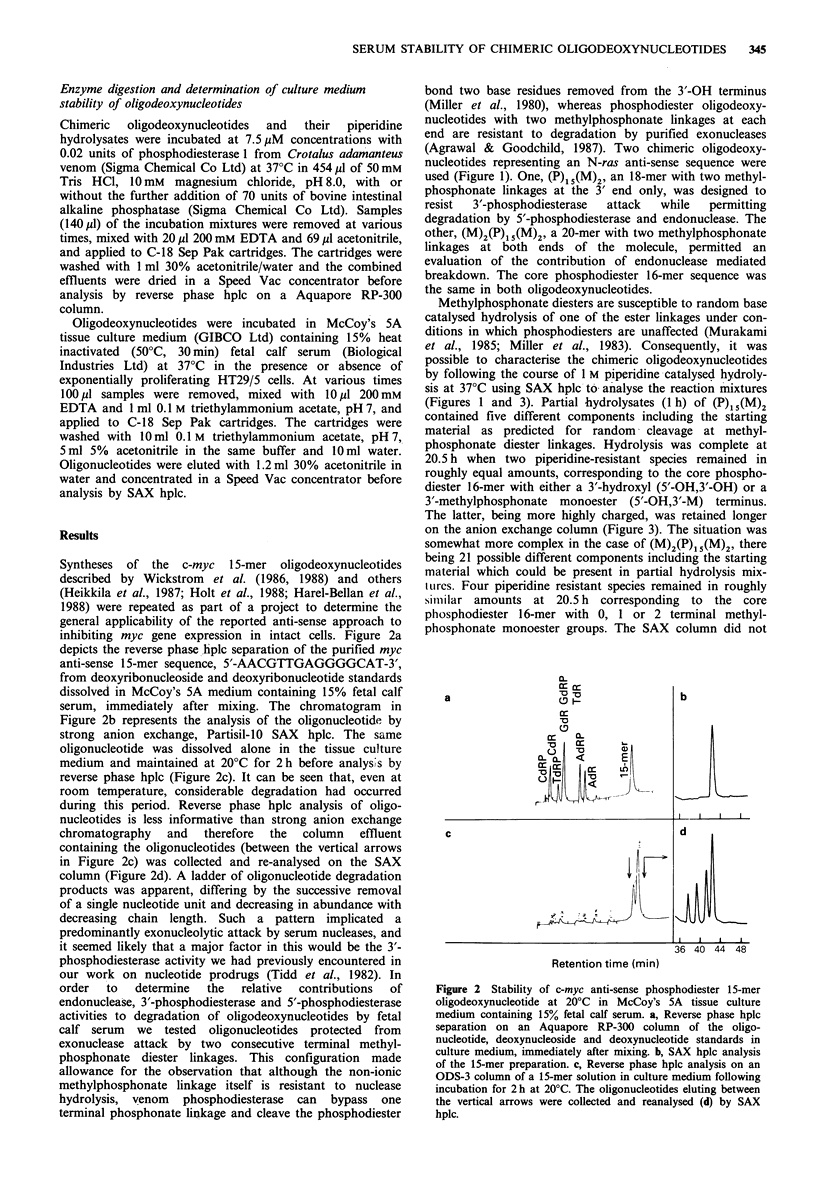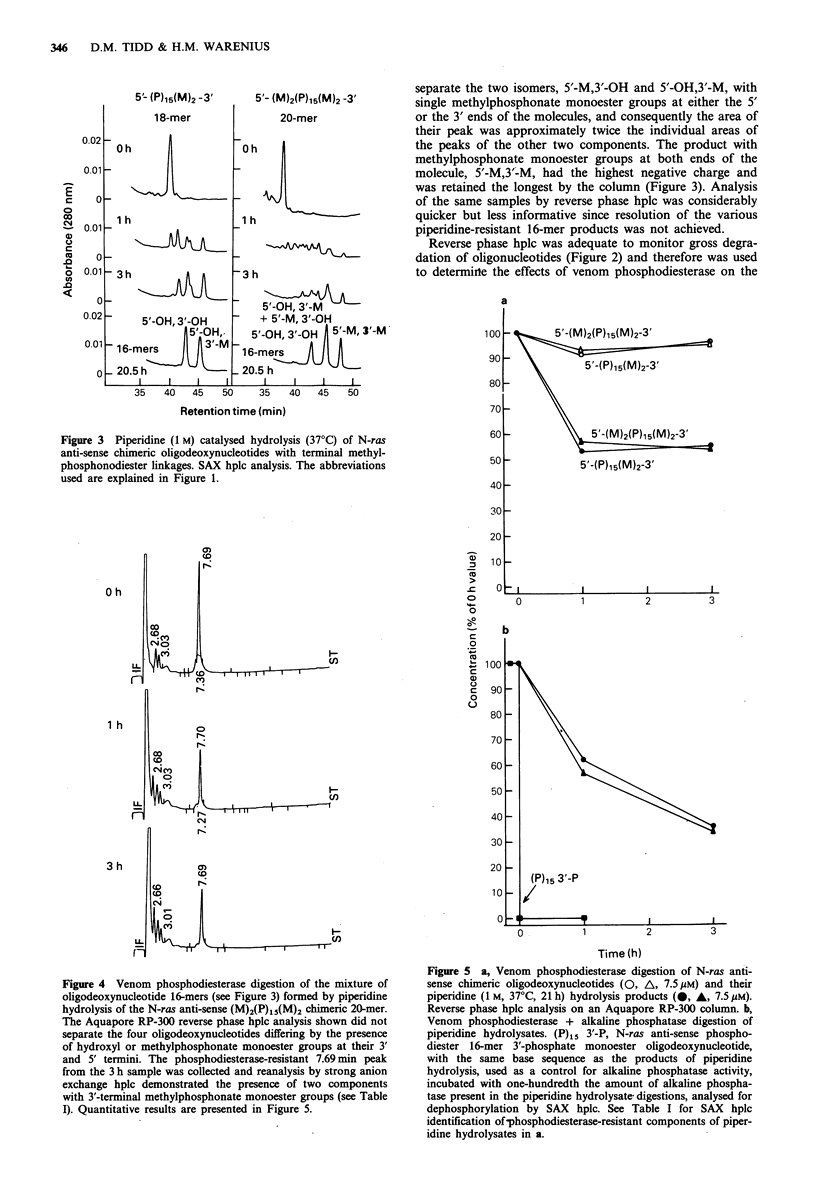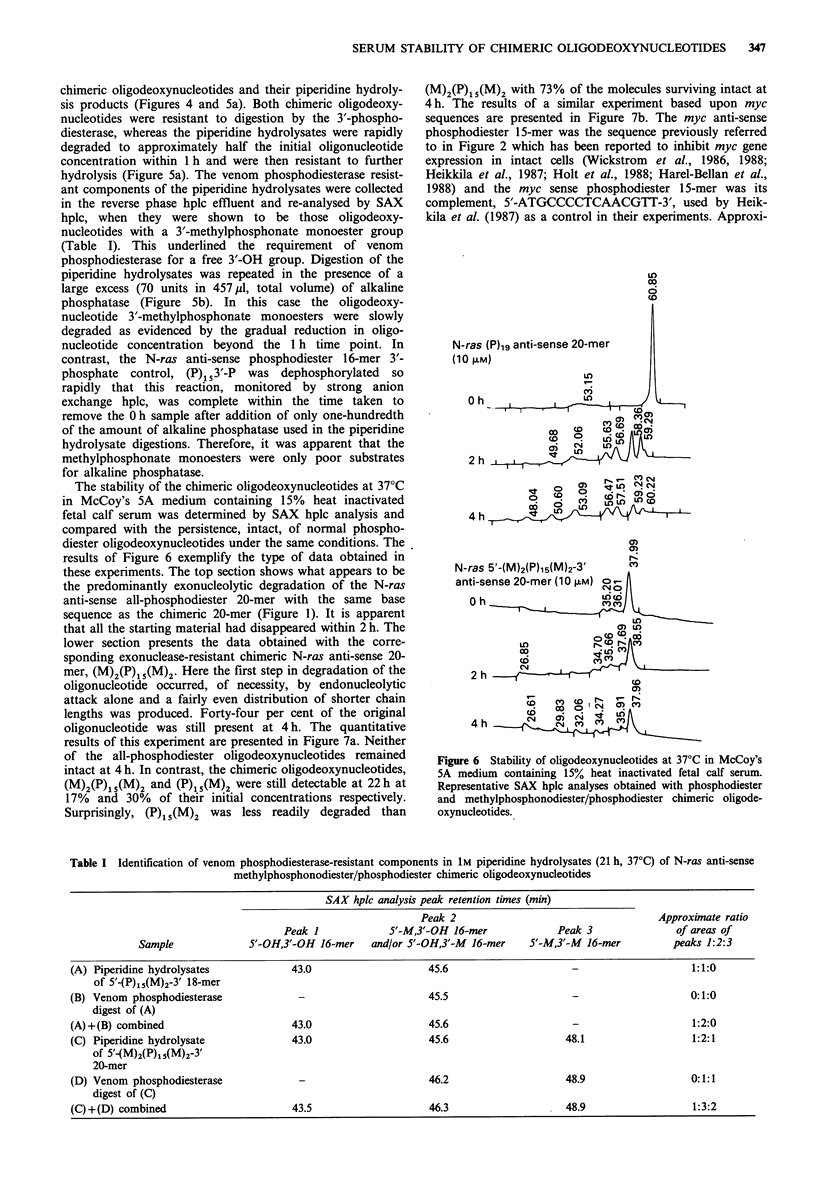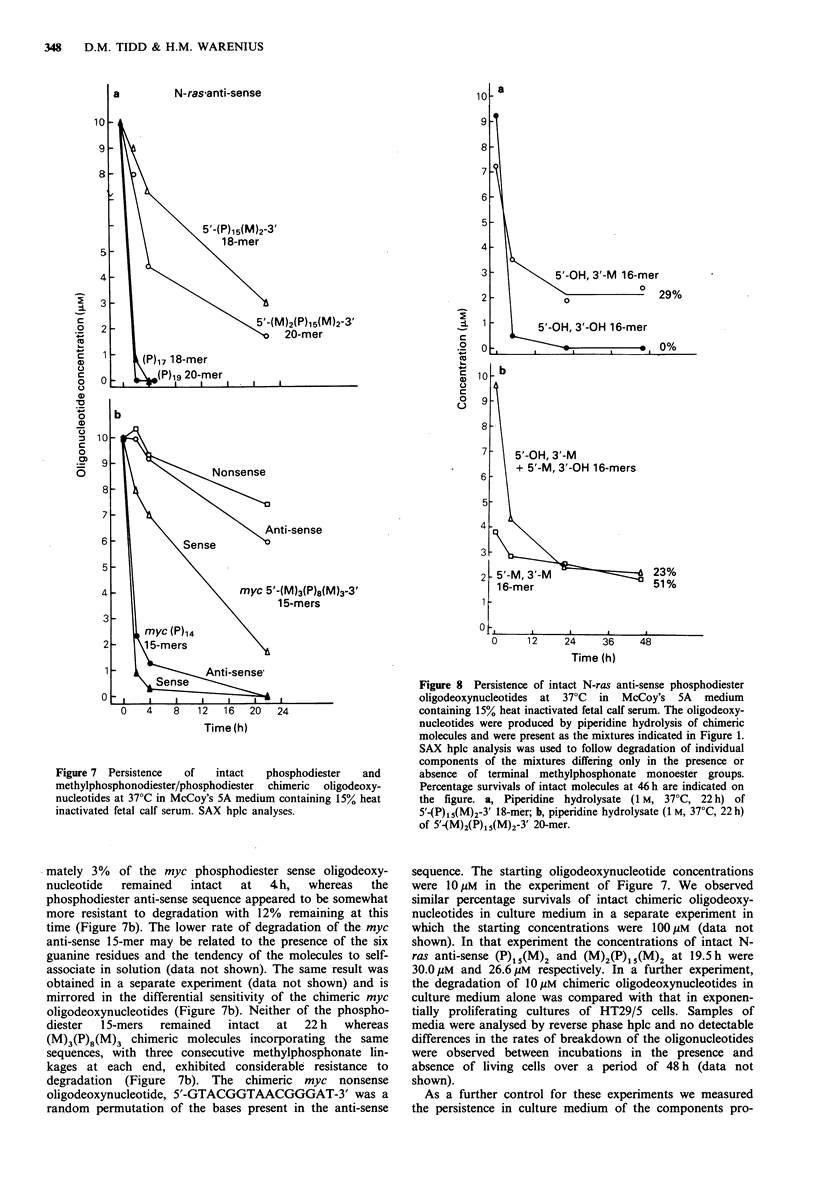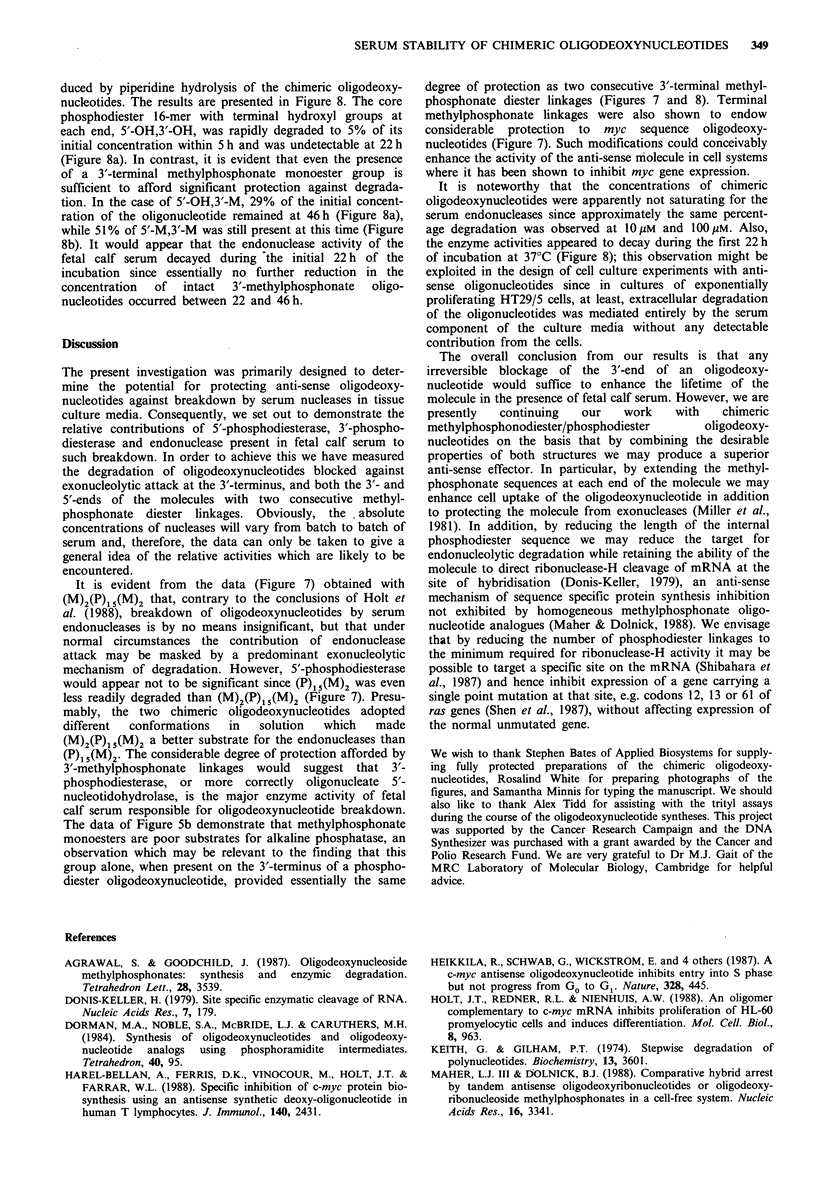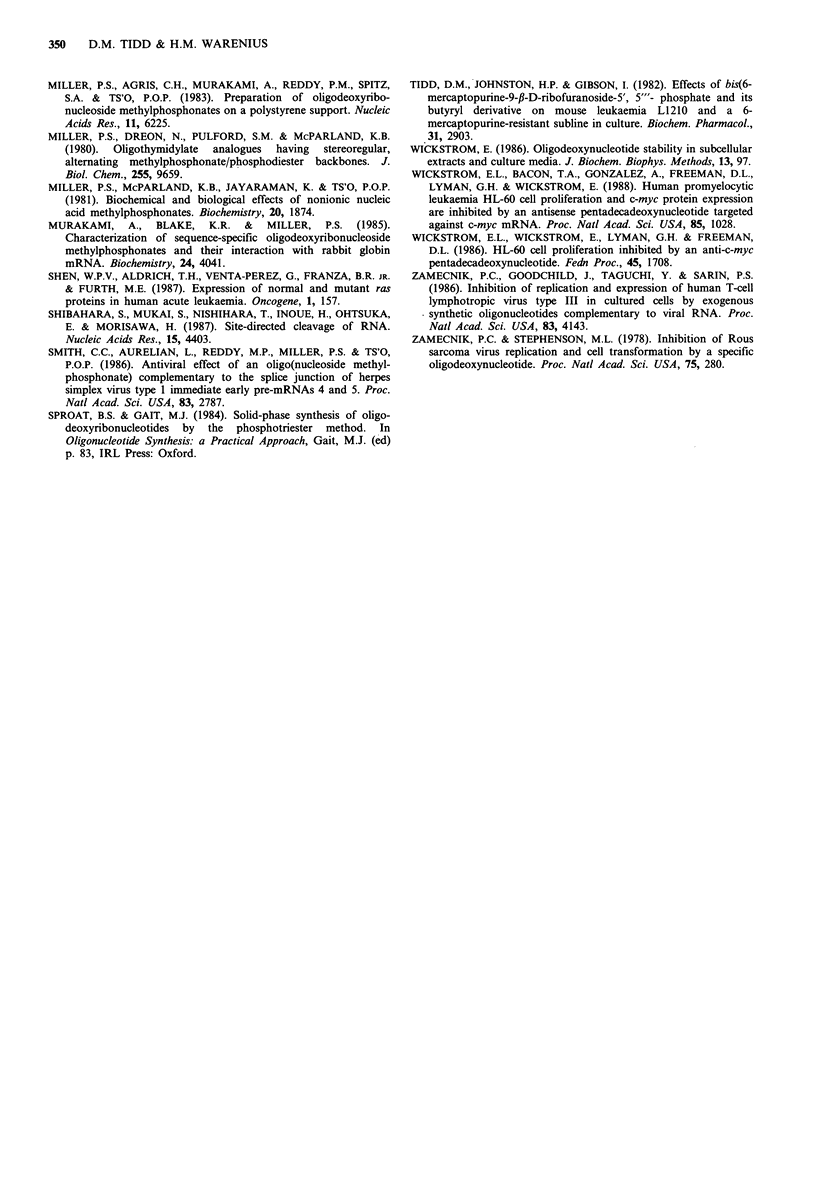# Partial protection of oncogene, anti-sense oligodeoxynucleotides against serum nuclease degradation using terminal methylphosphonate groups.

**DOI:** 10.1038/bjc.1989.283

**Published:** 1989-09

**Authors:** D. M. Tidd, H. M. Warenius

**Affiliations:** Cancer Research Campaign Department of Radiation Oncology, University of Liverpool, Clatterbridge Hospital, Bebington, Wirral, Merseyside, UK.

## Abstract

Under certain circumstances sequence-specific inhibition of gene expression may be achieved in intact cells using exogenous anti-sense oligodeoxynucleotides. The efficacy of this approach to investigating gene function is limited in part by the rapid serum nuclease mediated degradation of oligodeoxynucleotides in culture media. In order to determine the relative contributions of 3'-exonuclease, 5'-exonuclease and endonuclease activity in fetal calf serum to oligodeoxynucleotide destruction, we have tested chimeric N-ras anti-sense sequence molecules protected against exonuclease attack with terminal methylphosphonate diester linkages. An 18-mer with two methylphosphonate diester linkages at the 3'-terminus, a 20-mer with two methylphosphonate diester groups at both ends, and the 16-mer 3'-methylphosphonate monoester components of their respective piperidine hydrolysates were totally resistant to venom phosphodiesterase, whereas the 16-mer 3'-hydroxyl components of the hydrolysates were rapidly degraded. Both the chimeric oligodeoxynucleotides and 3'-methylphosphonate monoesters were considerably more stable than normal 3'-hydroxyl oligodeoxynucleotides at 37 degrees C in McCoy's 5A medium containing 15% heat inactivated fetal calf serum. Typically 20-30% of the former (initial concentration 10-100 microM) remained intact at 20 h as compared to the latter which were 88-100% degraded in 4 h and undetectable at 20 h. We conclude that a 3'-phosphodiesterase activity is a predominant nuclease responsible for oligodeoxynucleotide degradation by fetal calf serum, and that for cell culture studies, significant protection of oligodeoxynucleotides may be achieved by incorporating 3'-terminal methylphosphonate diester or even monoester end groups.


Br. J. Cancer (1989), 60, 343–350 © The Macmillan Press Ltd., 1989

### Partial protection of oncogene, anti-sense oligodeoxynucleotides against serum nuclease degradation using terminal methylphosphonate groups

D.M. Tidd & H.M. Warenius

*Cancer Research Campaign Department of Radiation Oncology, University of Liverpool, Clatterbridge Hospital, Bebington, Wirral, Merseyside L63 4JY, UK.*

**Summary** Under certain circumstances sequence-specific inhibition of gene expression may be achieved in intact cells using exogenous anti-sense oligodeoxynucleotides. The efficacy of this approach to investigating gene function is limited in part by the rapid serum nuclease mediated degradation of oligodeoxynucleotides in culture media. In order to determine the relative contributions of 3′-exonuclease, 5′-exonuclease and endonuclease activity in fetal calf serum to oligodeoxynucleotide destruction, we have tested chimeric N-*ras* anti-sense sequence molecules protected against exonuclease attack with terminal methylphosphonate diester linkages. An 18-mer with two methylphosphonate diester linkages at the 3′-terminus, a 20-mer with two methylphosphonate diester groups at both ends, and the 16-mer 3′-methylphosphonate monoester components of their respective piperidine hydrolysates were totally resistant to venom phosphodiesterase, whereas the 16-mer 3′-hydroxyl components of the hydrolysates were rapidly degraded. Both the chimeric oligodeoxynucleotides and 3′-methylphosphonate monoesters were considerably more stable than normal 3′-hydroxyl oligodeoxynucleotides at 37°C in McCoy's 5A medium containing 15% heat inactivated fetal calf serum. Typically 20–30% of the former (initial concentration 10–100 μM) remained intact at 20 h as compared to the latter which were 88–100% degraded in 4 h and undetectable at 20 h. We conclude that a 3′-phosphodiesterase activity is a predominant nuclease responsible for oligodeoxynucleotide degradation by fetal calf serum, and that for cell culture studies, significant protection of oligodeoxynucleotides may be achieved by incorporating 3′-terminal methylphosphonate diester or even monoester end groups.

Short synthetic anti-sense oligodeoxynucleotides with normal phosphodiester linkages have been reported to inhibit targeted gene expression when applied exogenously to intact cells in tissue culture (Zamecnik & Stephenson, 1978; Zamecnik *et al.*, 1986; Wickstrom *et al.*, 1986; Heikkila *et al.*, 1987; Harel-Bellan *et al.*, 1988; Holt *et al.*, 1988; Wickstrom *et al.*, 1988). However, the efficacy and general applicability of this approach to investigating oncogene or anti-oncogene function may be limited, in part, by the generally rapid serum nuclease mediated degradation of oligodeoxynucleotides in culture media. In contrast to our own experience, Wickstrom (1986) reported that a c-Ha-*ras* anti-sense 15-mer oligodeoxynucleotide was not detectably broken down in medium containing 5% fetal calf serum when incubated at 37°C for 2 h. However, a c-*myc* anti-sense 15-mer, which inhibited c-*myc* protein synthesis in intact HL-60 cells, underwent significant loss within 1 h in the culture supernatant containing 10% fetal calf serum and had virtually disappeared by 8 h (Wickstrom *et al.*, 1988). Others working on anti-sense oligodeoxynucleotide-induced inhibition of c-*myc* protein synthesis have carefully selected batches of fetal calf serum for low levels of nuclease activity at the outset by testing the degradation of $^{32}$P-labelled oligonucleotide (Harel-Bellan *et al.*, 1988; Holt *et al.*, 1988). Breakdown of c-*myc* sense and anti-sense 15-mers in media containing such selected serum was still appreciable and Holt *et al.* (1988) concluded that this occurred predominantly through exonuclease attack. It was not possible to determine from their data the actual relative contributions of 3′-phosphodiesterase, 5′-phosphodiesterase and endonuclease activities, although the latter appeared to be insignificant.

Zamecnik and co-workers have previously used oligodeoxynucleotides terminally blocked with isourea groups and with 3′-deoxythymidine on the assumption that these would be less susceptible to exonucleolytic enzymes present in the serum-containing incubation medium and within the cells (Zamecnik & Stephenson, 1978; Zamecnik *et al.*, 1986). However, the extent to which such modifications protected the oligodeoxynucleotides against degradation was not determined and, in the light of the variability in results obtained with HIV infected cells, these authors suggested that more work needed to be done with end-blocked oligonucleotides.

In the present work we have determined the relative extent to which 3′phosphodiesterase, 5′-phosphodiesterase and endonucleases present in fetal calf serum contribute to breakdown of oligodeoxynucleotides by using preparations of an N-*ras* anti-sense sequence (Figure 1) blocked at both the 5′ and 3′ ends or just the 3′ terminus with two methylphosphonate diester linkages (Smith *et al.*, 1986). In addition, piperidine catalysed hydrolysis of the parent chimeric molecules (Miller *et al.*, 1983; Murakami *et al.*, 1985) provided a convenient source of oligodeoxynucleotides with which to evaluate the protective effects of methylphosphonate monoester end groups (Figure 1). Our results suggest that a 3′-phosphodiesterase activity (oligonucleate 5′-nucleotidohydrolase) plays a predominant role in the breakdown of oligodeoxynucleotides by fetal calf serum and that significant increases in the half lives of intact molecules in tissue culture medium can be achieved by merely protecting the 3′ ends with methylphosphonate diester or even monoester groups.

### Materials and methods

### *Synthesis of oligodeoxynucleotides*

Methylphosphonodiester/phosphodiester chimeric oligodeoxynucleotides were very kindly assembled for us on an automatic DNA synthesiser by Stephen Bates of Applied Biosystems, Warrington, Cheshire, and were also synthesised in our own laboratory on an Applied Biosystems 381A Synthesizer, using a combination of methylphosphonamidite and β-cyanoethyl phosphoramidite chemistries, developed from the original work of Dorman *et al.* (1984). The oligonucleotides, in the fully protected, trityl-on, controlled pore glass support-bound form, were deprotected following the procedure for methylphosphonate oligonucleotide analogues (Maher & Dolnick, 1988). The products, carrying 5′-dimethoxytrityl groups as the sole remaining protecting functions, were dissolved in 5 ml 0.1 M

Correspondence: D.M. Tidd.
Received 28 October 1988, and in revised form, 28 April 1989.

Human N-ras gene sequence met thr glu tyr lys leu val ....
5'....A-T-G—A-C-T—G-A-G—T-A-C—A-A-A—C-T-G—G-T-G....3'
Codon no. 1 2 3 4 5 6 7

N-ras anti-sense chimeric oligodeoxynucleotides

3'....T/A/C—T-G-A—C-T-C—A-T-G—T-T-T—G-A-C....5' 5'-$(P)_{15}(M)_{2}$-3' 18-mer

3'....T/A/C—T-G-A—C-T-C—A-T-G—T-T-T—G-A-C/C/A....5' 5'-$(M)_{2}(P)_{15}(M)_{2}$-3' 20-mer

Components of 1M piperidine hydrolyzate of 5'-$(P)_{15}(M)_{2}$-3' 18-mer

3'....C—T-G-A—C-T-C—A-T-G—T-T-T—G-A-C....5' 5'-OH,3'-OH $(P)_{15}$ 16-mer

3'.../C—T-G-A—C-T-C—A-T-G—T-T-T—G-A-C....5' 5'-OH,3'-M $(P)_{15}$ 16-mer

Components of 1M piperidine hydrolyzate of 5'-$(M)_{2}(P)_{15}(M)_{2}$-3' 20-mer

3'....C—T-G-A—C-T-C—A-T-G—T-T-T—G-A-C....5' 5'-OH,3'-OH $(P)_{15}$ 16-mer

3'.../C—T-G-A—C-T-C—A-T-G—T-T-T—G-A-C....5' 5'-OH,3'-M $(P)_{15}$ 16-mer

3'....C—T-G-A—C-T-C—A-T-G—T-T-T—G-A-C/...5' 5'-M,3'-OH $(P)_{15}$ 16-mer

3'.../C—T-G-A—C-T-C—A-T-G—T-T-T—G-A-C/...5' 5'-M,3'-M $(P)_{15}$ 16-mer

**Figure 1** Structures of N-*ras* anti-sense methylphosphonodiester/phosphodiester chimeric oligodeoxynucleotides and their piperidine hydrolysis products. In the sequence representations, an internal / symbolises a methylphosphonate diester linkage and a terminal / signifies a methylphosphonate monoester group; an internal – represents a phosphodiester linkage. In the text the structures are referred to by a shorthand notation where P in parentheses with a subscript numeral represents the number of consecutive phosphodiester linkages, M in parentheses with a subscript numeral represents the number of consecutive methylphosphonate diester linkages, -OH represents a terminal hydroxyl, and -M a terminal methylphosphonate monoester group.

triethylammonium acetate, pH 7, containing 0.2% pyridine, to prevent premature detritylation, and were purified on C-18 Sep Pak cartridges (Waters Chromatography Division, Millipore (UK) Ltd). The cartridges were washed with 6 ml 0.2% pyridine/0.1 M triethylammonium acetate, pH 7, followed by 17 ml 15% acetonitrile/0.2% pyridine/0.1 M triethylammonium acetate, pH 7, to elute failure sequences. The dimethoxytrityl group was removed *in situ* with 10 ml 0.5% trifluoroacetic acid/water applied over a 15 min period, and the cartridges were washed with 10 ml 0.1 M triethylammonium acetate, pH 7, followed by 10 ml water. The products were eluted from the cartridges with 2 ml 30% acetonitrile/water and dried in a Savant Instruments Speed Vac Concentrator (Stratech Scientific Ltd). The oligonucleotide preparations were analysed by strong anion exchange hplc on a Partisil-10 SAX column (Whatman Ltd), temperature 45°C, 60 min gradient, 0.001–0.3 M potassium phosphate in 60% formamide/water, pH 6.8, 2 ml min$^{-1}$ (Sproat & Gait, 1984), and were shown to contain only minor quantities of failure sequences of shorter retention times than the single major peaks (see Figure 3). The oligonucleotides were deemed to be sufficiently pure for the present experiments and were used without further purification.

Phosphodiester oligodeoxyribonucleotides (trityl-on) were synthesised on an Applied Biosystems Model 381A DNA Synthesizer and were deprotected in concentrated ammonia solution for 8 h at 60°C. Following deprotection the oligodeoxynucleotides were purified on C-18 Sep Pak cartridges by the same procedure as that described for the chimeric oligodeoxynucleotides. The products were analysed by SAX hplc, and reverse phase hplc on Partisil-10 ODS-3 (Whatman Ltd) and Aquapore RP-300 (Applied Biosystems Ltd) columns using a 30 min gradient from 0 to 30% acetonitrile in 0.1 M triethylammonium acetate, pH 7, at a flow rate of 1.5 ml min$^{-1}$. No impurities were detected in these preparations. The N-*ras* anti-sense phosphodiester oligodeoxyribonucleotide 16-mer, corresponding to the phosphodiester portion of the chimeric oligodeoxynucleotides (Figure 1), and carrying a 3′-terminal phosphate monoester group, was synthesised via an intermediate 17-mer with a 3′-terminal ribonucleoside, uridine, using a uridine-derivatised controlled pore glass support (Peninsula Laboratories Europe Ltd). The uridine was subsequently removed by periodate oxidation and β-elimination to yield the oligodeoxyribonucleotide 3′-phosphate derivative (Keith & Gilham, 1974).

### *Piperidine catalysed hydrolysis of chimeric oligodeoxynucleotides*

Chimeric oligodeoxynucleotides were incubated at 37°C in 1 M solutions of piperidine. At the end of the incubation periods the piperidine was either neutralised with an equivalent amount of 6 N hydrochloric acid or removed by evaporation in a Speed Vac concentrator, followed by co-evaporation twice with water to eliminate trace residues of the base. Hydrolysed samples were analysed by SAX hplc, and by reverse phase hplc on an Aquapore RP-300 column using a 20 min gradient of 5–50% acetonitrile/0.1 M triethylammonium acetate, pH 7, at a flow rate of 2 ml min$^{-1}$.

*Enzyme digestion and determination of culture medium stability of oligodeoxynucleotides*

Chimeric oligodeoxynucleotides and their piperidine hydrolysates were incubated at 7.5 $\mu$M concentrations with 0.02 units of phosphodiesterase I from *Crotalus adamanteus* venom (Sigma Chemical Co Ltd) at 37°C in 454 $\mu$l of 50 mM Tris HCl, 10 mM magnesium chloride, pH 8.0, with or without the further addition of 70 units of bovine intestinal alkaline phosphatase (Sigma Chemical Co Ltd). Samples (140 $\mu$l) of the incubation mixtures were removed at various times, mixed with 20 $\mu$l 200 mM EDTA and 69 $\mu$l acetonitrile, and applied to C-18 Sep Pak cartridges. The cartridges were washed with 1 ml 30% acetonitrile/water and the combined effluents were dried in a Speed Vac concentrator before analysis by reverse phase hplc on a Aquapore RP-300 column.

Oligodeoxynucleotides were incubated in McCoy's 5A tissue culture medium (GIBCO Ltd) containing 15% heat inactivated (50°C, 30 min) fetal calf serum (Biological Industries Ltd) at 37°C in the presence or absence of exponentially proliferating HT29/5 cells. At various times 100 $\mu$l samples were removed, mixed with 10 $\mu$l 200 mM EDTA and 1 ml 0.1 M triethylammonium acetate, pH 7, and applied to C-18 Sep Pak cartridges. The cartridges were washed with 10 ml 0.1 M triethylammonium acetate, pH 7, 5 ml 5% acetonitrile in the same buffer and 10 ml water. Oligonucleotides were eluted with 1.2 ml 30% acetonitrile in water and concentrated in a Speed Vac concentrator before analysis by SAX hplc.

### Results

Syntheses of the c-*myc* 15-mer oligodeoxynucleotides described by Wickstrom *et al.* (1986, 1988) and others (Heikkila *et al.*, 1987; Holt *et al.*, 1988; Harel-Bellan *et al.*, 1988) were repeated as part of a project to determine the general applicability of the reported anti-sense approach to inhibiting *myc* gene expression in intact cells. Figure 2a depicts the reverse phase hplc separation of the purified *myc* anti-sense 15-mer sequence, 5′-AACGTTGAGGGGCAT-3′, from deoxyribonucleoside and deoxyribonucleotide standards dissolved in McCoy's 5A medium containing 15% fetal calf serum, immediately after mixing. The chromatogram in Figure 2b represents the analysis of the oligonucleotide by strong anion exchange, Partisil-10 SAX hplc. The same oligonucleotide was dissolved alone in the tissue culture medium and maintained at 20°C for 2 h before analysis by reverse phase hplc (Figure 2c). It can be seen that, even at room temperature, considerable degradation had occurred during this period. Reverse phase hplc analysis of oligonucleotides is less informative than strong anion exchange chromatography and therefore the column effluent containing the oligonucleotides (between the vertical arrows in Figure 2c) was collected and re-analysed on the SAX column (Figure 2d). A ladder of oligonucleotide degradation products was apparent, differing by the successive removal of a single nucleotide unit and decreasing in abundance with decreasing chain length. Such a pattern implicated a predominantly exonucleolytic attack by serum nucleases, and it seemed likely that a major factor in this would be the 3′-phosphodiesterase activity we had previously encountered in our work on nucleotide prodrugs (Tidd *et al.*, 1982). In order to determine the relative contributions of endonuclease, 3′-phosphodiesterase and 5′-phosphodiesterase activities to degradation of oligodeoxynucleotides by fetal calf serum we tested oligonucleotides protected from exonuclease attack by two consecutive terminal methylphosphonate diester linkages. This configuration made allowance for the observation that although the non-ionic methylphosphonate linkage itself is resistant to nuclease hydrolysis, venom phosphodiesterase can bypass one terminal phosphonate linkage and cleave the phosphodiester bond two base residues removed from the 3′-OH terminus (Miller *et al.*, 1980), whereas phosphodiester oligodeoxynucleotides with two methylphosphonate linkages at each end are resistant to degradation by purified exonucleases (Agrawal & Goodchild, 1987). Two chimeric oligodeoxynucleotides representing an N-*ras* anti-sense sequence were used (Figure 1). One, $(P)_{15}(M)_2$, an 18-mer with two methylphosphonate linkages at the 3′ end only, was designed to resist 3′-phosphodiesterase attack while permitting degradation by 5′-phosphodiesterase and endonuclease. The other, $(M)_2(P)_{15}(M)_2$, a 20-mer with two methylphosphonate linkages at both ends of the molecule, permitted an evaluation of the contribution of endonuclease mediated breakdown. The core phosphodiester 16-mer sequence was the same in both oligodeoxynucleotides.

Methylphosphonate diesters are susceptible to random base catalysed hydrolysis of one of the ester linkages under conditions in which phosphodiesters are unaffected (Murakami *et al.*, 1985; Miller *et al.*, 1983). Consequently, it was possible to characterise the chimeric oligodeoxynucleotides by following the course of 1 M piperidine catalysed hydrolysis at 37°C using SAX hplc to analyse the reaction mixtures (Figures 1 and 3). Partial hydrolysates (1 h) of $(P)_{15}(M)_2$ contained five different components including the starting material as predicted for random cleavage at methylphosphonate diester linkages. Hydrolysis was complete at 20.5 h when two piperidine-resistant species remained in roughly equal amounts, corresponding to the core phosphodiester 16-mer with either a 3′-hydroxyl (5′-OH,3′-OH) or a 3′-methylphosphonate monoester (5′-OH,3′-M) terminus. The latter, being more highly charged, was retained longer on the anion exchange column (Figure 3). The situation was somewhat more complex in the case of $(M)_2(P)_{15}(M)_2$, there being 21 possible different components including the starting material which could be present in partial hydrolysis mixtures. Four piperidine resistant species remained in roughly similar amounts at 20.5 h corresponding to the core phosphodiester 16-mer with 0, 1 or 2 terminal methylphosphonate monoester groups. The SAX column did not

**Figure 2** Stability of c-*myc* anti-sense phosphodiester 15-mer oligodeoxynucleotide at 20°C in McCoy's 5A tissue culture medium containing 15% fetal calf serum. **a**, Reverse phase hplc separation on an Aquapore RP-300 column of the oligonucleotide, deoxynucleoside and deoxynucleotide standards in culture medium, immediately after mixing. **b**, SAX hplc analysis of the 15-mer preparation. **c**, Reverse phase hplc analysis on an ODS-3 column of a 15-mer solution in culture medium following incubation for 2 h at 20°C. The oligonucleotides eluting between the vertical arrows were collected and reanalysed (**d**) by SAX hplc.

separate the two isomers, 5′-M,3′-OH and 5′-OH,3′-M, with single methylphosphonate monoester groups at either the 5′ or the 3′ ends of the molecules, and consequently the area of their peak was approximately twice the individual areas of the peaks of the other two components. The product with methylphosphonate monoester groups at both ends of the molecule, 5′-M,3′-M, had the highest negative charge and was retained the longest by the column (Figure 3). Analysis of the same samples by reverse phase hplc was considerably quicker but less informative since resolution of the various piperidine-resistant 16-mer products was not achieved.

Reverse phase hplc was adequate to monitor gross degradation of oligonucleotides (Figure 2) and therefore was used to determine the effects of venom phosphodiesterase on the

**Figure 3** Piperidine (1 M) catalysed hydrolysis (37°C) of N-*ras* anti-sense chimeric oligodeoxynucleotides with terminal methylphosphonodiester linkages. SAX hplc analysis. The abbreviations used are explained in Figure 1.

**Figure 4** Venom phosphodiesterase digestion of the mixture of oligodeoxynucleotide 16-mers (see Figure 3) formed by piperidine hydrolysis of the N-*ras* anti-sense $(M)_2(P)_{15}(M)_2$ chimeric 20-mer. The Aquapore RP-300 reverse phase hplc analysis shown did not separate the four oligodeoxynucleotides differing by the presence of hydroxyl or methylphosphonate monoester groups at their 3′ and 5′ termini. The phosphodiesterase-resistant 7.69 min peak from the 3 h sample was collected and reanalysis by strong anion exchange hplc demonstrated the presence of two components with 3′-terminal methylphosphonate monoester groups (see Table I). Quantitative results are presented in Figure 5.

**Figure 5** **a**, Venom phosphodiesterase digestion of N-*ras* anti-sense chimeric oligodeoxynucleotides (○, △, 7.5 μM) and their piperidine (1 M, 37°C, 21 h) hydrolysis products (●, ▲, 7.5 μM). Reverse phase hplc analysis on an Aquapore RP-300 column. **b**, Venom phosphodiesterase + alkaline phosphatase digestion of piperidine hydrolysates. $(P)_{15}$ 3′-P, N-*ras* anti-sense phosphodiester 16-mer 3′-phosphate monoester oligodeoxynucleotide, with the same base sequence as the products of piperidine hydrolysis, used as a control for alkaline phosphatase activity, incubated with one-hundredth the amount of alkaline phosphatase present in the piperidine hydrolysate digestions, analysed for dephosphorylation by SAX hplc. See Table I for SAX hplc identification of phosphodiesterase-resistant components of piperidine hydrolysates in **a**.

chimeric oligodeoxynucleotides and their piperidine hydrolysis products (Figures 4 and 5a). Both chimeric oligodeoxynucleotides were resistant to digestion by the 3′-phosphodiesterase, whereas the piperidine hydrolysates were rapidly degraded to approximately half the initial oligonucleotide concentration within 1 h and were then resistant to further hydrolysis (Figure 5a). The venom phosphodiesterase resistant components of the piperidine hydrolysates were collected in the reverse phase hplc effluent and re-analysed by SAX hplc, when they were shown to be those oligodeoxynucleotides with a 3′-methylphosphonate monoester group (Table I). This underlined the requirement of venom phosphodiesterase for a free 3′-OH group. Digestion of the piperidine hydrolysates was repeated in the presence of a large excess (70 units in 457 μl, total volume) of alkaline phosphatase (Figure 5b). In this case the oligodeoxynucleotide 3′-methylphosphonate monoesters were slowly degraded as evidenced by the gradual reduction in oligonucleotide concentration beyond the 1 h time point. In contrast, the N-*ras* anti-sense phosphodiester 16-mer 3′-phosphate control, $(P)_{15}$3′-P was dephosphorylated so rapidly that this reaction, monitored by strong anion exchange hplc, was complete within the time taken to remove the 0 h sample after addition of only one-hundredth of the amount of alkaline phosphatase used in the piperidine hydrolysate digestions. Therefore, it was apparent that the methylphosphonate monoesters were only poor substrates for alkaline phosphatase.

The stability of the chimeric oligodeoxynucleotides at 37°C in McCoy's 5A medium containing 15% heat inactivated fetal calf serum was determined by SAX hplc analysis and compared with the persistence, intact, of normal phosphodiester oligodeoxynucleotides under the same conditions. The results of Figure 6 exemplify the type of data obtained in these experiments. The top section shows what appears to be the predominantly exonucleolytic degradation of the N-*ras* anti-sense all-phosphodiester 20-mer with the same base sequence as the chimeric 20-mer (Figure 1). It is apparent that all the starting material had disappeared within 2 h. The lower section presents the data obtained with the corresponding exonuclease-resistant chimeric N-*ras* anti-sense 20-mer, $(M)_2(P)_{15}(M)_2$. Here the first step in degradation of the oligonucleotide occurred, of necessity, by endonucleolytic attack alone and a fairly even distribution of shorter chain lengths was produced. Forty-four per cent of the original oligonucleotide was still present at 4 h. The quantitative results of this experiment are presented in Figure 7a. Neither of the all-phosphodiester oligodeoxynucleotides remained intact at 4 h. In contrast, the chimeric oligodeoxynucleotides, $(M)_2(P)_{15}(M)_2$ and $(P)_{15}(M)_2$ were still detectable at 22 h at 17% and 30% of their initial concentrations respectively. Surprisingly, $(P)_{15}(M)_2$ was less readily degraded than $(M)_2(P)_{15}(M)_2$ with 73% of the molecules surviving intact at 4 h. The results of a similar experiment based upon *myc* sequences are presented in Figure 7b. The *myc* anti-sense phosphodiester 15-mer was the sequence previously referred to in Figure 2 which has been reported to inhibit *myc* gene expression in intact cells (Wickstrom *et al.*, 1986, 1988; Heikkila *et al.*, 1987; Holt *et al.*, 1988; Harel-Bellan *et al.*, 1988) and the *myc* sense phosphodiester 15-mer was its complement, 5′-ATGCCCCTCAACGTT-3′, used by Heikkila *et al.* (1987) as a control in their experiments. Approxi-

**Figure 6** Stability of oligodeoxynucleotides at 37°C in McCoy's 5A medium containing 15% heat inactivated fetal calf serum. Representative SAX hplc analyses obtained with phosphodiester and methylphosphonodiester/phosphodiester chimeric oligodeoxynucleotides.

**Table I** Identification of venom phosphodiesterase-resistant components in 1M piperidine hydrolysates (21 h, 37°C) of N-*ras* anti-sense methylphosphonodiester/phosphodiester chimeric oligodeoxynucleotides

| | *SAX hplc analysis peak retention times (min)* | | | |
|---|---|---|---|---|
| *Sample* | *Peak 1 5′-OH,3′-OH 16-mer* | *Peak 2 5′-M,3′-OH 16-mer and/or 5′-OH,3′-M 16-mer* | *Peak 3 5′-M,3′-M 16-mer* | *Approximate ratio of areas of peaks 1:2:3* |
| (A) Piperidine hydrolysates of 5′-$(P)_{15}(M)_2$-3′ 18-mer | 43.0 | 45.6 | – | 1:1:0 |
| (B) Venom phosphodiesterase digest of (A) | – | 45.5 | – | 0:1:0 |
| (A)+(B) combined | 43.0 | 45.6 | – | 1:2:0 |
| (C) Piperidine hydrolysate of 5′-$(M)_2(P)_{15}(M)_2$-3′ 20-mer | 43.0 | 45.6 | 48.1 | 1:2:1 |
| (D) Venom phosphodiesterase digest of (C) | – | 46.2 | 48.9 | 0:1:1 |
| (C)+(D) combined | 43.5 | 46.3 | 48.9 | 1:3:2 |

**Figure 7** Persistence of intact phosphodiester and methylphosphonodiester/phosphodiester chimeric oligodeoxynucleotides at 37°C in McCoy's 5A medium containing 15% heat inactivated fetal calf serum. SAX hplc analyses.

**Figure 8** Persistence of intact N-*ras* anti-sense phosphodiester oligodeoxynucleotides at 37°C in McCoy's 5A medium containing 15% heat inactivated fetal calf serum. The oligodeoxynucleotides were produced by piperidine hydrolysis of chimeric molecules and were present as the mixtures indicated in Figure 1. SAX hplc analysis was used to follow degradation of individual components of the mixtures differing only in the presence or absence of terminal methylphosphonate monoester groups. Percentage survivals of intact molecules at 46 h are indicated on the figure. **a**, Piperidine hydrolysate (1 M, 37°C, 22 h) of 5'-$(P)_{15}(M)_2$-3' 18-mer; **b**, piperidine hydrolysate (1 M, 37°C, 22 h) of 5'-$(M)_2(P)_{15}(M)_2$-3' 20-mer.

mately 3% of the *myc* phosphodiester sense oligodeoxynucleotide remained intact at 4 h, whereas the phosphodiester anti-sense sequence appeared to be somewhat more resistant to degradation with 12% remaining at this time (Figure 7b). The lower rate of degradation of the *myc* anti-sense 15-mer may be related to the presence of the six guanine residues and the tendency of the molecules to self-associate in solution (data not shown). The same result was obtained in a separate experiment (data not shown) and is mirrored in the differential sensitivity of the chimeric *myc* oligodeoxynucleotides (Figure 7b). Neither of the phosphodiester 15-mers remained intact at 22 h whereas $(M)_3(P)_8(M)_3$ chimeric molecules incorporating the same sequences, with three consecutive methylphosphonate linkages at each end, exhibited considerable resistance to degradation (Figure 7b). The chimeric *myc* nonsense oligodeoxynucleotide, 5'-GTACGGTAACGGGAT-3' was a random permutation of the bases present in the anti-sense sequence. The starting oligodeoxynucleotide concentrations were 10 μM in the experiment of Figure 7. We observed similar percentage survivals of intact chimeric oligodeoxynucleotides in culture medium in a separate experiment in which the starting concentrations were 100 μM (data not shown). In that experiment the concentrations of intact N-*ras* anti-sense $(P)_{15}(M)_2$ and $(M)_2(P)_{15}(M)_2$ at 19.5 h were 30.0 μM and 26.6 μM respectively. In a further experiment, the degradation of 10 μM chimeric oligodeoxynucleotides in culture medium alone was compared with that in exponentially proliferating cultures of HT29/5 cells. Samples of media were analysed by reverse phase hplc and no detectable differences in the rates of breakdown of the oligonucleotides were observed between incubations in the presence and absence of living cells over a period of 48 h (data not shown).

As a further control for these experiments we measured the persistence in culture medium of the components produced by piperidine hydrolysis of the chimeric oligodeoxynucleotides. The results are presented in Figure 8. The core phosphodiester 16-mer with terminal hydroxyl groups at each end, 5′-OH,3′-OH, was rapidly degraded to 5% of its initial concentration within 5 h and was undetectable at 22 h (Figure 8a). In contrast, it is evident that even the presence of a 3′-terminal methylphosphonate monoester group is sufficient to afford significant protection against degradation. In the case of 5′-OH,3′-M, 29% of the initial concentration of the oligonucleotide remained at 46 h (Figure 8a), while 51% of 5′-M,3′-M was still present at this time (Figure 8b). It would appear that the endonuclease activity of the fetal calf serum decayed during the initial 22 h of the incubation since essentially no further reduction in the concentration of intact 3′-methylphosphonate oligonucleotides occurred between 22 and 46 h.

### Discussion

The present investigation was primarily designed to determine the potential for protecting anti-sense oligodeoxynucleotides against breakdown by serum nucleases in tissue culture media. Consequently, we set out to demonstrate the relative contributions of 5′-phosphodiesterase, 3′-phosphodiesterase and endonuclease present in fetal calf serum to such breakdown. In order to achieve this we have measured the degradation of oligodeoxynucleotides blocked against exonucleolytic attack at the 3′-terminus, and both the 3′- and 5′-ends of the molecules with two consecutive methylphosphonate diester linkages. Obviously, the absolute concentrations of nucleases will vary from batch to batch of serum and, therefore, the data can only be taken to give a general idea of the relative activities which are likely to be encountered.

It is evident from the data (Figure 7) obtained with $(M)_2(P)_{15}(M)_2$ that, contrary to the conclusions of Holt *et al.* (1988), breakdown of oligodeoxynucleotides by serum endonucleases is by no means insignificant, but that under normal circumstances the contribution of endonuclease attack may be masked by a predominant exonucleolytic mechanism of degradation. However, 5′-phosphodiesterase would appear not to be significant since $(P)_{15}(M)_2$ was even less readily degraded than $(M)_2(P)_{15}(M)_2$ (Figure 7). Presumably, the two chimeric oligodeoxynucleotides adopted different conformations in solution which made $(M)_2(P)_{15}(M)_2$ a better substrate for the endonucleases than $(P)_{15}(M)_2$. The considerable degree of protection afforded by 3′-methylphosphonate linkages would suggest that 3′-phosphodiesterase, or more correctly oligonucleate 5′-nucleotidohydrolase, is the major enzyme activity of fetal calf serum responsible for oligodeoxynucleotide breakdown. The data of Figure 5b demonstrate that methylphosphonate monoesters are poor substrates for alkaline phosphatase, an observation which may be relevant to the finding that this group alone, when present on the 3′-terminus of a phosphodiester oligodeoxynucleotide, provided essentially the same degree of protection as two consecutive 3′-terminal methylphosphonate diester linkages (Figures 7 and 8). Terminal methylphosphonate linkages were also shown to endow considerable protection to *myc* sequence oligodeoxynucleotides (Figure 7). Such modifications could conceivably enhance the activity of the anti-sense molecule in cell systems where it has been shown to inhibit *myc* gene expression.

It is noteworthy that the concentrations of chimeric oligodeoxynucleotides were apparently not saturating for the serum endonucleases since approximately the same percentage degradation was observed at 10 μM and 100 μM. Also, the enzyme activities appeared to decay during the first 22 h of incubation at 37°C (Figure 8); this observation might be exploited in the design of cell culture experiments with anti-sense oligonucleotides since in cultures of exponentially proliferating HT29/5 cells, at least, extracellular degradation of the oligonucleotides was mediated entirely by the serum component of the culture media without any detectable contribution from the cells.

The overall conclusion from our results is that any irreversible blockage of the 3′-end of an oligodeoxynucleotide would suffice to enhance the lifetime of the molecule in the presence of fetal calf serum. However, we are presently continuing our work with chimeric methylphosphonodiester/phosphodiester oligodeoxynucleotides on the basis that by combining the desirable properties of both structures we may produce a superior anti-sense effector. In particular, by extending the methylphosphonate sequences at each end of the molecule we may enhance cell uptake of the oligodeoxynucleotide in addition to protecting the molecule from exonucleases (Miller *et al.*, 1981). In addition, by reducing the length of the internal phosphodiester sequence we may reduce the target for endonucleolytic degradation while retaining the ability of the molecule to direct ribonuclease-H cleavage of mRNA at the site of hybridisation (Donis-Keller, 1979), an anti-sense mechanism of sequence specific protein synthesis inhibition not exhibited by homogeneous methylphosphonate oligonucleotide analogues (Maher & Dolnick, 1988). We envisage that by reducing the number of phosphodiester linkages to the minimum required for ribonuclease-H activity it may be possible to target a specific site on the mRNA (Shibahara *et al.*, 1987) and hence inhibit expression of a gene carrying a single point mutation at that site, e.g. codons 12, 13 or 61 of *ras* genes (Shen *et al.*, 1987), without affecting expression of the normal unmutated gene.

We wish to thank Stephen Bates of Applied Biosystems for supplying fully protected preparations of the chimeric oligodeoxynucleotides, Rosalind White for preparing photographs of the figures, and Samantha Minnis for typing the manuscript. We should also like to thank Alex Tidd for assisting with the trityl assays during the course of the oligodeoxynucleotide syntheses. This project was supported by the Cancer Research Campaign and the DNA Synthesizer was purchased with a grant awarded by the Cancer and Polio Research Fund. We are very grateful to Dr M.J. Gait of the MRC Laboratory of Molecular Biology, Cambridge for helpful advice.

### References

AGRAWAL, S. & GOODCHILD, J. (1987). Oligodeoxynucleoside methylphosphonates: synthesis and enzymic degradation. *Tetrahedron Lett.*, **28**, 3539.

DONIS-KELLER, H. (1979). Site specific enzymatic cleavage of RNA. *Nucleic Acids Res.*, **7**, 179.

DORMAN, M.A., NOBLE, S.A., McBRIDE, L.J. & CARUTHERS, M.H. (1984). Synthesis of oligodeoxynucleotides and oligodeoxynucleotide analogs using phosphoramidite intermediates. *Tetrahedron*, **40**, 95.

HAREL-BELLAN, A., FERRIS, D.K., VINOCOUR, M., HOLT, J.T. & FARRAR, W.L. (1988). Specific inhibition of c-*myc* protein biosynthesis using an antisense synthetic deoxy-oligonucleotide in human T lymphocytes. *J. Immunol.*, **140**, 2431.

HEIKKILA, R., SCHWAB, G., WICKSTROM, E. and 4 others (1987). A c-*myc* antisense oligodeoxynucleotide inhibits entry into S phase but not progress from $G_0$ to $G_1$. *Nature*, **328**, 445.

HOLT, J.T., REDNER, R.L. & NIENHUIS, A.W. (1988). An oligomer complementary to c-*myc* mRNA inhibits proliferation of HL-60 promyelocytic cells and induces differentiation. *Mol. Cell. Biol.*, **8**, 963.

KEITH, G. & GILHAM, P.T. (1974). Stepwise degradation of polynucleotides. *Biochemistry*, **13**, 3601.

MAHER, L.J. III & DOLNICK, B.J. (1988). Comparative hybrid arrest by tandem antisense oligodeoxyribonucleotides or oligodeoxyribonucleoside methylphosphonates in a cell-free system. *Nucleic Acids Res.*, **16**, 3341.

MILLER, P.S., AGRIS, C.H., MURAKAMI, A., REDDY, P.M., SPITZ, S.A. & TS'O, P.O.P. (1983). Preparation of oligodeoxyribonucleoside methylphosphonates on a polystyrene support. *Nucleic Acids Res.*, **11**, 6225.

MILLER, P.S., DREON, N., PULFORD, S.M. & McPARLAND, K.B. (1980). Oligothymidylate analogues having stereoregular, alternating methylphosphonate/phosphodiester backbones. *J. Biol. Chem.*, **255**, 9659.

MILLER, P.S., McPARLAND, K.B., JAYARAMAN, K. & TS'O, P.O.P. (1981). Biochemical and biological effects of nonionic nucleic acid methylphosphonates. *Biochemistry*, **20**, 1874.

MURAKAMI, A., BLAKE, K.R. & MILLER, P.S. (1985). Characterization of sequence-specific oligodeoxyribonucleoside methylphosphonates and their interaction with rabbit globin mRNA. *Biochemistry*, **24**, 4041.

SHEN, W.P.V., ALDRICH, T.H., VENTA-PEREZ, G., FRANZA, B.R. JR. & FURTH, M.E. (1987). Expression of normal and mutant *ras* proteins in human acute leukaemia. *Oncogene*, **1**, 157.

SHIBAHARA, S., MUKAI, S., NISHIHARA, T., INOUE, H., OHTSUKA, E. & MORISAWA, H. (1987). Site-directed cleavage of RNA. *Nucleic Acids Res.*, **15**, 4403.

SMITH, C.C., AURELIAN, L., REDDY, M.P., MILLER, P.S. & TS'O, P.O.P. (1986). Antiviral effect of an oligo(nucleoside methylphosphonate) complementary to the splice junction of herpes simplex virus type 1 immediate early pre-mRNAs 4 and 5. *Proc. Natl Acad. Sci. USA*, **83**, 2787.

SPROAT, B.S. & GAIT, M.J. (1984). Solid-phase synthesis of oligodeoxyribonucleotides by the phosphotriester method. In *Oligonucleotide Synthesis: a Practical Approach*, Gait, M.J. (ed) p. 83, IRL Press: Oxford.

TIDD, D.M., JOHNSTON, H.P. & GIBSON, I. (1982). Effects of *bis*(6-mercaptopurine-9-β-D-ribofuranoside-5′, 5‴- phosphate and its butyryl derivative on mouse leukaemia L1210 and a 6-mercaptopurine-resistant subline in culture. *Biochem. Pharmacol.*, **31**, 2903.

WICKSTROM, E. (1986). Oligodeoxynucleotide stability in subcellular extracts and culture media. *J. Biochem. Biophys. Methods*, **13**, 97.

WICKSTROM, E.L., BACON, T.A., GONZALEZ, A., FREEMAN, D.L., LYMAN, G.H. & WICKSTROM, E. (1988). Human promyelocytic leukaemia HL-60 cell proliferation and c-*myc* protein expression are inhibited by an antisense pentadecadeoxynucleotide targeted against c-*myc* mRNA. *Proc. Natl Acad. Sci. USA*, **85**, 1028.

WICKSTROM, E.L., WICKSTROM, E., LYMAN, G.H. & FREEMAN, D.L. (1986). HL-60 cell proliferation inhibited by an anti-c-*myc* pentadecadeoxynucleotide. *Fedn Proc.*, **45**, 1708.

ZAMECNIK, P.C., GOODCHILD, J., TAGUCHI, Y. & SARIN, P.S. (1986). Inhibition of replication and expression of human T-cell lymphotropic virus type III in cultured cells by exogenous synthetic oligonucleotides complementary to viral RNA. *Proc. Natl Acad. Sci. USA*, **83**, 4143.

ZAMECNIK, P.C. & STEPHENSON, M.L. (1978). Inhibition of Rous sarcoma virus replication and cell transformation by a specific oligodeoxynucleotide. *Proc. Natl Acad. Sci. USA*, **75**, 280.